# Effects of Olive Pomace and Spice Extracts on Performance and Antioxidant Function in Broiler Chickens

**DOI:** 10.3390/ani15060808

**Published:** 2025-03-12

**Authors:** Fernando Sevillano, Marta Blanch, Jose J. Pastor, Miguel Angel Ibáñez, David Menoyo

**Affiliations:** 1 Departamento de Producción Agraria, ETS Ingeniería Agronómica, Alimentaria y de Biosistemas, Universidad Politécnica de Madrid, 28040 Madrid, Spain; f.sevillano@alumnos.upm.es; 2 Innovation Division, Lucta S. A., UAB Research Park, Edifici Eureka, 08193 Bellaterra, Spain; marta.blanch@lucta.com (M.B.); jose.pastor@lucta.com (J.J.P.); 3 Departamento de Economía Agraria, Estadística y Gestión de Empresas, ETS Ingeniería Agronómica, Alimentaria y de Biosistemas, Universidad Politécnica de Madrid, 28040 Madrid, Spain; miguel.ibanez@upm.es

**Keywords:** broiler chicken, olive extract, spices extract, antioxidant function

## Abstract

This research aimed to evaluate the effects of an olive pomace extract (OE) and a fat-encapsulated extract composed of a blend of oleoresins from *Capsicum* sp., black pepper, and ginger (SPICY) on broiler chicken performance and antioxidant function. In total, 640 1-day-old male chicks were randomly assigned to five experimental diets (eight replicates/treatment, 16 birds/replicate). Diets included a basal diet with no added vitamin E (NC), NC plus 100 ppm of vitamin E (PC), NC plus 1250 ppm of OE, NC plus 250 ppm of (SPICY), and NC plus 1250 ppm OE plus 250 ppm of SPICY (SPIOE). Phytogenic additives were supplied by Lucta S.A., Spain. Compared to the NC, the PC significantly (*p* < 0.05) increased ADG from 8 to 14 days of age, with both OE and SPICY showing intermediate values between both controls. At the end of this trial, at 35 days of age, a significant (*p* < 0.05) increase in plasma GPx activity was observed in PC-fed birds compared to the NC, with no effects of malonyl dialdehyde (MDA) and total antioxidant capacity. Birds fed the OE and SPICY displayed intermediate values of GPx activity compared to both controls. The expression of heat shock protein 70 (HSP70) and glutathione S-Transferase Alpha 4 (GSTA4) was significantly lower (*p* < 0.05) in the jejunal mucosa of birds fed the OE compared to the NC. Moreover, the expression of HSP70 was significantly lower (*p* < 0.05) in birds fed the OE compared to SPICY but was not significantly different compared to the blend of both extracts (SPIOE). In conclusion, OE and SPICY were useful in maintaining growth performance in no vit E-supplemented diets, particularly in the case of OE mediated by its antioxidant action through HSP70.

## Simple Summary

In recent years, there has been an increased interest in the use of phytogenic additives with antioxidant capacity to partially or fully replace the synthetic vitamin E (vit E) that is added to broiler feeds for this purpose. However, research into phytogenic antioxidant activity mechanisms is required to determine whether they can replace or act synergistically with vit E. In the present study, we tested the antioxidant properties of an olive pomace extract (OE) and a spice-based extract (SPICY), with capsaicin as the main active compound, compared to two control diets with basal (10 ppm supplied by the raw materials) or high (100 ppm) of added vit E. Results highlight the potential use of OE and SPICY extracts in diets with low vit E levels to maintain growth performance, especially in the starter phase, and shed light on their mechanisms of action.

## 1. Introduction

Broiler chickens destined for commercial meat production nowadays have a very high performance potential due to improvements in genetic selection, management, and nutritional practices [1,2]. However, this potential bind to intensive production makes these birds more prone to oxidative stress, which might negatively affect the birds’ health, performance, and meat quality [1]. To face this oxidative stress, it is important to enhance the antioxidant defenses of the birds by supplementing their diet with antioxidant-rich substances such as α-tocopherol, ascorbate, selenium, magnesium, and zinc [1]. Vitamin E (vit E, α-tocopherol) is the most used and effective antioxidant in poultry feeds [3]. The National Research Council [4] establishes a minimum of 10 UI/kg of vit E in broiler feeds to avoid nutritional deficiencies. However, many poultry integrators recommend adding vit E above physiological levels (e.g., 50 to 100 ppm) because it provides extra benefits to the birds by enhancing the antioxidant and immune response to oxidative stress, resulting in improved growth performance under common stress situations [5]. However, the synthetic nature of this added vit E and its high price has led to the search for cost-effective and natural antioxidant alternatives [6]. These might include natural sources of vit E coming from plants or insects [7,8], which have shown good bioavailability and positive effects in poultry traits [9,10], as well as phytogenic additives.

Phytogenics are an enormous group of compounds coming from different parts of the plants that can be used as feed additives due to their beneficial proprieties (e.g., antioxidant, biocide, anti-inflammatory, or digestibility enhancers) [11]. Among these proprieties, the antioxidant capacity of many phytogenics has been strongly proven. These compounds are rich in molecules with proven bioactivity, among which are phenolic compounds and flavonoids [12], capable of stopping or slowing down oxidative reactions by directly scavenging free radicals but also reacting with oxygen or combining with catalytic metal ions to create complexes that make them inactive [9]. Extracts from spiced plants such as red pepper, black pepper, or ginger have phenolic compounds and alkaloids (e.g., capsaicin, caffeic acid, kaempferol, quercetin, gingerol, shogaol, etc.) with known free radical scavenging mechanisms of action [13]. Another interesting group of phytogenic additives is agrifood by-products. Olive pomace, a by-product obtained after olive oil extraction, is rich in polyphenols and terpenoids with a strong antioxidant function that can directly inactivate free radicals [14,15]. In addition to this free radical scavenging activity, recent studies have shown that phytogenic additives can improve the antioxidant response of birds by activating the nuclear factor erythroid 2-related factor 2 (Nrf2), the transcription factor that regulates the expression of antioxidant enzymes [16].

The beneficial effects of spice extracts in animal nutrition have been studied in several trials. The anti-microbial, antioxidant, and anti-inflammatory activity of capsaicin (8-methyl-N-vanilla base-6-nonene amide) has been proven [17,18], in addition to its benefits in gastrointestinal function and productive traits [19,20]. Other spice compounds, such as piperine or gingerol, have antioxidant, anti-inflammatory, and gut protection properties and have been tested in poultry [21]. A blend of encapsulated *Capsicum*, black pepper, and ginger extracts was recently tested in broilers by Herrero-Encinas et al. [22], who found that this additive could positively affect the performance during the first week of age of the birds, improve nutrient digestibility, and enhance the antioxidant response.

The anti-inflammatory and antioxidant capacity of olive pomace extracts has been proven in many studies [23,24,25,26,27]. Among olive polyphenols, hydroxytyrosol (4-(2-Hydroxyethyl) benzene-1,2-diol; HT), the main polyphenol in olive pomace, has proven to display higher antioxidant capacity than vit E or butylated hydroxytoluene by Le Tutour and Guedon [28]. Within this antioxidant capacity, HT seems to improve the oxidative stress status of broilers, understood as a response to many common stressors (environmental, handling, or feeding) [29]. Related to this antioxidant capacity, olive compounds seem to decrease lipid peroxidation in the liver of the broilers [30].

All these studies clearly show that additives based on phytogenics with antioxidant properties might be an interesting natural alternative as substitutes for supplemented vit E. However, research into their antioxidant action mechanisms is required to ascertain if they can function in place of or in concert with vit E. Thus, the main purpose of this study was to evaluate the mechanisms of antioxidant action of two phytogenic feed additives an olive pomace extract (OE) and a fat encapsulated extract composed of a blend of oleoresins from *Capsicum* sp., black pepper and ginger (SPICY) compared to vit E in broiler chickens.

## 2. Materials and Methods

The procedures used in this research were approved by the Animal Ethics Committee of the Universidad Politécnica de Madrid (Permission No. 2022-062) and were in compliance with the Spanish Guidelines for the Care and Use of Animals in Research [31].

### 2.1. Birds and Husbandry

This research was conducted at the experimental facilities of the Universidad Politécnica de Madrid (Department of Agricultural Production, ETSIAAB). A total of 640 one-day-old male broiler chicks (Cobb 500; 44.8 ± 0.55 g) were randomly allotted to 40 floor pens (0.96 × 1.5 m) in groups of 16 birds per pen, separated into similar rooms (20 pens per room). Pen bedding consisted of cereal straw pellets. The pens were equipped with 4 nipple drinkers and a hopper feeder. During the first week of age, additional first-age feeders were used. Water and feed were provided for ad libitum consumption throughout this experiment. The environmental conditions of the rooms were controlled automatically according to commercial practices [32]. The temperature of the rooms was set at 33 °C during the first week of age, and then it was reduced by 2 °C per week until reaching 24 °C at the end of this experiment. Birds received a light program of 23 L:1 D during the first week of age, and light time was progressively reduced to 18 L:6 D, keeping it until the end of this trial.

### 2.2. Experimental Design and Diets

This experiment was a randomized complete block design with the room as a block and 5 experimental diets distributed across 40 pens, 20 in each room, thus 8 replicates per treatment in total, 4 replicates of each treatment per room. The feeding program consisted of two periods: starter (from 1 to 21 days, crumbled) and grower (from 22 to 35 days, pellets). Basal starter and grower diets were formulated to meet the nutrient requirements of the broilers, according to FEDNA [33]. Table 1 shows the ingredients and the composition of the basal diets (starter and grower). The basal diets were supplemented as follows to obtain the 5 experimental treatments. Starter feeds: a negative control with no additives and no added vit E (NC), a positive control with 100 ppm of added vit E (PC), the NC supplemented with 1250 ppm of olive pomace extract (OE), the NC supplemented with 250 ppm of the spicy extract (SPICY), and the NC supplemented with 250 ppm of SPICY plus 1250 ppm of OE (SPIOE). The diets during the growth phase maintained the same concentrations of vit E and OE as the starter, but the SPICY treatments decreased the concentration to 125 ppm. Phytogenic additives were added on top of feeds and supplied by Lucta S.A. (Madrid, Spain). The OE consisted of an olive pomace extract standardized to contain a minimum of 2% total triterpenes and 0.3% polyphenols. The SPICY is a fat-encapsulated extract composed of a blend of oleoresins from *Capsicum* sp., black pepper, and ginger, with capsaicin as the main active compound. The concentrations of OE and SPICY in the feeds were based on our previous studies [22,23,24]. The experimental feeds were manufactured at the Institute of Agrifood Research and Technology (IRTA; Mas de Bover, Constanti, Spain). Raw materials provided approximately 10 ppm of vit E to the feeds.

### 2.3. Laboratory Analysis

Representative samples of the diets were ground using a laboratory mill (Retsch Model Z-I, Stuttgart, Germany) fitted with a 0.75 mm screen and analyzed for moisture by oven-drying (method 930.15), ash by a muffle furnace (method 942.05), and nitrogen by combustion (method 968.06) using a Leco equipment (FP-528, Leco Corporation, St. Joseph, MI, USA), as indicated by AOAC International [34]. The gross energy of the feed was determined using an adiabatic bomb calorimeter (model 6400, Parr Instrument Company, Moline, IL, USA). Vit E concentration in the feed was determined by HPLC according to the 2000/45/EC directive. The ether extract was determined by an ANKOM XT10 extractor (ANKOM Technology, Macedon, NY, USA), as indicated in AOAC method 920.39.

### 2.4. Growth Performance and Sampling

Feed disappearance and body weight of the birds were recorded by box at 7, 14, 21, 28, and 35 days of age, and mortality was recorded as produced. From these data, the average daily gain (ADG), average daily feed intake (ADFI), and feed conversion ratio (FCR) were determined by period (1 to 7 days, 8 to 14 days, 15 to 21 days, 22 to 28 days, and 29 to 35 days of age), phase (starter, from 1 to 21 days; and grower, from 22 to 35 days of age) and cumulatively (0 to 35 days of age). At the end of this experiment (day 35), two birds per box were randomly selected and euthanized in the CO_2_ atmosphere. A sample of blood was collected in anticoagulant vacuum tubes (BD Vacutainer^®^, Plymouth, UK) and centrifuged. Once the plasma was separated, samples were frozen at −80 °C. A small piece of the right lobe of the liver was also collected and frozen at −80 °C. A piece of the jejunum was cut and scraped, and the resulting mucosa was also frozen at −80 °C.

### 2.5. Plasma TBARs and Total Antioxidant Capacity

Thiobarbituric acid reactive substances (TBARs) in plasma samples of broilers were measured as the reaction products of malondialdehyde (MDA) with thiobarbituric acid, as described elsewhere [35]. Total antioxidant capacity (TAC) was determined using a commercial kit following the instruction of the manufacturer (antioxidant assay kit, Cayman Chemical Kit catalogue no. 709001; Cayman Chemical, Ann Arbor, MI, USA). The kit is based on the ability of aqueous and lipid-soluble antioxidants present in the sample to inhibit the oxidation of ABTS (2, 2’-azino-bis (3-ethylbenzthiazoline-6-sulphonicacid)) by metmyoglobin, which is monitored by reading absorbance at 750 nm and it is quantified as millimolar Trolox equivalents.

### 2.6. Plasma Enzyme Activity and α-Tocopherol Content

Plasma enzyme activities were determined using commercial kits and following the instruction of the manufacturer, particularly glutathione peroxidase activity (GPx, Cayman Chemical Kit catalog no. 703102; Cayman Chemical, Ann Arbor, MI, USA), superoxide dismutase activity (SOD, Cayman Chemical Kit catalog no. 706002; Cayman Chemical, Ann Arbor, MI, USA), and catalase activity (CAT, Cayman Chemical Kit catalog no. 707002; Cayman Chemical, Ann Arbor, MI, USA). The content of α-tocopherol in plasma was measured by HPLC, according to Rey et al. [36].

### 2.7. Gene Expression Analysis

Total RNA was extracted from 50 mg of jejunum mucosa and 25 mg of liver using TRIzol reagent (Invitrogen, Carlsbad, CA, USA). Tissue was desegregated with a mixer mill MM-400 (Retsch, Stuttgart, Germany) and purified using a PureLink RNA Minikit (Thermo Fisher Scientific, Waltham, MA, USA). To prevent genomic DNA contamination, an “in column” DNase step was performed using the RNAse-Free DNase Set (Quiagen, Australia). The yield and quality of the extracted RNA were measured using an Epoch spectrophotometer (BioTek, Winoosky, VT, USA) with a Take3 micro-volume Plate (BioTek, Santa Barbara, CA, USA). Reverse transcription of around 2400 ng of extracted RNA was performed with the SuperScript VILO Master Mix (Invitrogen, Carlsbad, CA, USA). The quantitative real-time PCR (qRT-PCR) analysis was performed in a 7300 Real-Time PCR System (Applied Biosystems, Foster City, CA, USA) with already tested and published primer conditions (Table 2). Samples were analyzed in duplicate using the adequate quantity of each primer, DNAse Free water, and SYBR Green Master Mix (Applied Biosystems, Foster City, CA, USA). Analysis of the results was based on the Cycle threshold (Ct) using the automatic Ct provided by the qPCR software (7300 System SDS Software, Version 1.4; Applied Biosystems). Gene expression was normalized with the geometric mean of the two reference genes (Beta-actin and Ubiquitin).

### 2.8. Statistical Analysis

For all the statistical analyses, SAS software 9.04 was used (SAS Institute, Cary, NC, USA), with treatments as fixed effects and the room as random block effect. Growth performance, plasma enzyme activity, plasma α-tocopherol content, and gene expression were analyzed by an ANOVA test (mean, ± SEM), a Dunnet test comparing the phytogenic treatments (OE, SPICY, SPIOE) and the two controls (NC and PC), and a Tukey test comparing phytogenic treatments with each other. Significant differences were set with *p*-values < 0.05, and tendencies were set with *p*-values between 0.05 and 0.10.

For genes displaying efficiencies different from 2 (E ≠ 2), cycle threshold (Ct) values were adjusted according to the model described by Steibel [44]. The above-mentioned statistical tests were used with the expression of target genes normalized with the geometric mean of the reference genes β-actin and Ubiquitin, according to Pfaffl et al. [45]. The standard error (SE) was used to recalculate the lower and upper 95% confidence intervals for each fold change.

## 3. Results

### 3.1. Growth Performance

Table 3 summarizes the growth performance of the birds during this trial. No significant differences were observed in performance among dietary treatments during the first week of age. However, from 8 to 14 days of age, the ADG was significantly lower (*p* < 0.05) in birds fed the NC compared to those birds fed the PC. This lower ADG resulted in a trend to lower BW at 21 days of age in birds fed the NC compared to PC (1026 g vs. 1063 g respectively; *p* = 0.0742). In the global period, from 0 to 21 days of age, the ADG tended (*p* = 0.0715) to be lower in birds fed the NC, 46.7 vs. 48.5 g/d for NC and PC, respectively. In the second period, from 22 to 35 days of age, no significant differences were observed among experimental treatments on performance or feed intake.

No significant differences were observed in performance between birds fed the phytogenic additives and the NC or PC (controls) in either of the two periods. Among phytogenic additives, the only notable tendency (*p* = 0.051) was to decrease feed intake during the first week of age with the combination of SPICY and OE compared to the SPICY diet alone. Global mortality was 3.2% and was not affected by dietary treatments.

### 3.2. Plasma TBARs and Total Antioxidant Capacity

No significant differences were observed in TBARs or TAC between birds fed the NC and the PC (Table 4). Also, no significant differences were observed between phytogenic additives and the NC or PC (controls). When comparing the different additives, a trend (*p* = 0.0507) was found in plasma TBARs between OE and SPICY (0.243 vs. 0.418 μM MDA, respectively).

### 3.3. Plasma Enzyme Activity and α-Tocopherol Content

The activity of CAT and SOD in plasma was not affected by dietary means. However, the activity of GPx was significantly higher (*p* < 0.05) in birds fed the PC compared to the NC (Table 4). The activity of this enzyme was similar in birds fed the PC, OE, and SPICY, but compared to PC, birds fed the SPIOE diet showed a significantly lower (*p* < 0.05) GPx activity (Table 4). As expected, α-tocopherol concentration was significantly higher (*p* < 0.001) in the plasma of birds fed the PC compared to the rest of the diets. Broilers fed the NC or the diets with phytogenic additives showed similar α-tocopherol levels (Table 4).

### 3.4. Gene Expression

There were no significant differences in gene expression in the jejunum mucosa between PC and NC (Table 5). Birds fed the OE group displayed significant downregulation of HSP70 compared to NC (*p* = 0.013) and SPICY (*p* = 0.0241). Also, GSTA4 was downregulated in the OE (*p* < 0.01) and SPIOE (*p* = 0.0335) groups compared to NC. In the liver, no significant differences were observed by dietary means (Table 6).

## 4. Discussion

### 4.1. Growth Performance

It is well known that under optimal rearing conditions, vit E concentration in the feed has little effect on productive indices once the broiler needs for vit E are satisfied [46]. Nonetheless, broilers raised for commercial purposes encounter a series of stressors that may surpass their antioxidant capacity. In these situations, an increase in vit E content in the feed may improve the performance of the animals [47]. This is especially critical in young birds, in which their vit E recycling system does not seem to be as effective as in adult birds [48,49]. During the early days of broiler life, vit E is essential for the adequate development of the chick, which must face oxidative stress; at the same time, they grow up fast [50]. Moreover, young birds have low stocks of vit E, which rapidly decreases during the first 14 days of life, and supplementation with this vit in the feed above needs can lessen this reduction [5]. In the present study, broilers fed the PC diet showed higher ADG compared to those fed the NC in the second week of the trial. This effect was reflected in the BW and the ADG at 21 d of age, as PC birds tended to have higher weight and ADG compared to NC in the starter phase (from 0 to 21 d). Our data align with those of Panda et al. [51], who observed better weight gain at 21 d of age in chickens fed diets containing 30 ppm of supplemented vit E compared to a control group supplemented with 10 ppm. Also, Chae et al. [52] reported better weight gain at 21 d of age in birds fed supplemented with vit E compared to a no-supplement diet. These data highlight the relevance of vit E supplementation in the starting phase. In the second period, from 22 to 35 days of age, no significant differences were observed between birds fed the NC and the PC diets. This is in line with what was observed in a recent meta-analysis confirming that during the grower phase, when there are no stress situations for the animal, an increase in vit E in the diet does not seem to have repercussions on productive parameters [46].

In the present study, there were no significant differences in performance between the chickens fed with the PC and those provided with phytogenic additives during the starter and grower periods. Recent reviews show the potential of using phytogenic additives in poultry feeds on productive parameters because of their functional properties related to digestive, antioxidant, and immune systems [26]. The phytogenic additives used in this study, which are based on spices or olive oil by-products, have generally been shown to have some favorable benefits on animal productivity, both under normal but particularly under challenging conditions [22,53]. Although the present study did not present challenging or stressful situations for the animals, the additives were useful in maintaining good productivity, especially during the first phase of this study, showing an intermediate behavior between the PC and the NC. Compared to lipid-soluble antioxidants like vit E, water-soluble antioxidants such as polyphenols may be advantageous for young chicks with low lipase enzyme activity. Therefore, the inclusion of OE and SPICY extracts might be useful to maintain productivity in the starter phase in diets with low vit E supplementation.

### 4.2. α-Tocopherol (vit E) Plasma Concentrations

The concentrations of vit E in plasma were significantly higher in animals fed with the PC. This was somewhat expected given the positive correlation between dietary and plasma vit E concentrations [54,55,56]. Despite the observed differences in vit E plasma concentrations, no significant differences were observed between dietary treatments on TTPA expression in the liver. The TTPA gene encodes a key protein for the control of plasma tocopherol concentration. Our results are in line with those obtained by Rengaraj et al. [57], who did not observe significant differences in TTPA expression in the liver of chickens fed with dietary concentrations of vit E that ranged from 0 to 100 ppm. As suggested by Rengaraj et al. [57], the discrepant results between liver TTPA expression and serum α-tocopherol concentrations might be because of post-transcriptional events that need further research.

It has been observed that polyphenols can positively affect the oxidative processes of vit E and, thus, influence its recycling [58]. But, negative interactions have also been reported regarding intestinal absorption with polyphenols affecting vit E transporters such as scavenger receptor class B type I and NPC1-like transporter 1 [59]. In the present study, the animals fed with the NC and the phytogenic additives showed similar concentrations of vit E. This aligns with our previous studies in which we also did not observe changes in plasma or liver vit E concentrations in chickens fed with additives like those used in this study [22,60]. This suggests that under our experimental conditions, phytogenic additives have no effect on plasma vit E concentrations.

### 4.3. Antioxidant Function

It is generally accepted that increased vit E supplementation above physiological levels (e.g., 50 to 100 mg/kg) gives the birds additional advantages by strengthening their immune system and antioxidant defenses against oxidative stress [5]. The main role of vit E as an antioxidant is to scavenge chain-carrying lipid peroxyl radicals to break the chain propagation of lipid peroxidation [61]. Studies in which birds are subjected to oxidative stress through heat stress or the use of unsaturated lipid sources compared to saturated ones demonstrate positive effects of dietary vit E on lipid peroxidation levels, measured as MDA [47]. In our study, MDA concentrations and TAC did not significantly differ between the PC and the NC, despite the observed variations in plasma vit E concentrations. This might suggest that the environmental and nutritional conditions in this study did not considerably raise oxidative stress and lipid peroxidation. According to Panda et al. [51], broilers given increased vit E concentrations had improved performance and antioxidant status, which was linked to lower MDA concentrations and higher GPx activity in plasma without observing changes in CAT activity. In the present study, we observed a similar response of vit E on plasma antioxidant enzyme activities, with birds fed the PC showing higher GPx activity than those fed the NC without changes in CAT or SOD activities. In addition to its direct activity as a radical scavenger, vit E can improve the antioxidant capacity indirectly by upregulating the expression of genes involved in glutathione synthesis [62]. An increase in glutathione concentrations because of dietary vit E has been reported in broilers [48]. Given that glutathione is a substrate for the GPx activity, it is plausible that our data reflect this indirect antioxidant action of vit E. Lipid hydroperoxides and hydrogen peroxide are reductively inactivated by the enzyme GPx, preventing their harmful accumulation. Therefore, although the study conditions did not involve high oxidative stress to detect changes in performance or MDA plasma concentrations, it might be expected that birds fed the high dose of vit E were more protected from oxidative stress due to the increased GPx activity.

It has been reported that Vit E can indirectly affect the expression of regulatory genes such as Nrf2, the master transcription factor regulating the expression of the antioxidant enzymes, including GPx, SOD, or CAT [63]. In the present study, no significant differences were observed between the PC and the NC in the expression of target genes related to antioxidant response in the liver and jejunum. This included Nrf2 and his regulated genes GPx, SOD, CAT, GSTA4, and GSTM2, or other genes related to oxidative stress, such as HSP70 and NOX. Similar to our findings, Korošec et al. [64] reported no significant differences in liver Nrf2 expression of chickens fed 73.8 compared to those fed 8.48 mg/kg of α-tocopherol. However, they reported significant downregulation of GSTM2 and GSTA3 in the liver. The lack of effect of vit E on jejunum and liver antioxidant gene expression coincides with the minimal effects observed in plasma, except the GPx activity, which was significantly higher in plasma of birds fed the PC. The discrepancies between GPx activity in tissues and plasma may be because, in plasma, the assay kit used measures the activity of all the glutathione-dependent peroxidases, while in tissues, we measure the expression of GPx1. In avian species, the GPx includes at least eight members with different properties and functions, and GPx1 is one of the four Se-dependent forms [65]. The discrepant effects observed may also be due to the varying susceptibility of the different tissues to stress [66].

There is increasing interest in the use of plant-based additives as natural antioxidants in broiler nutrition [67]. Although, in recent years, many studies have been conducted observing positive effects on various parameters related to oxidative stress, there is still a gap in knowledge regarding the possible mechanisms of action and synergies of these additives. Phytogenic compounds like polyphenols can help to maintain the antioxidant balance of the birds by directly scavenging the free radicals or inducing antioxidant enzymes by activating Nrf2 [67]. In the same way, the antioxidant capacity of capsaicin might be due to radical scavenging or via Nrf2 [68]. In the present study, MDA concentrations and the total antioxidant capacity in plasma were similar in animals fed the phytogenic additives and the PC and NC. However, birds fed the OE tended to have lower MDA values than those fed the SPICY. Despite this, birds fed OE and SPICY displayed an intermediate GPx enzyme activity between the PC and the NC, as no significant differences were observed between them. Like vit E, olive polyphenols and capsaicin have been reported to increase glutathione concentrations [68,69]. The hormetic principle has been proposed as one of the possible ways to explain the antioxidant effects of phytogenics. By inducing mild cellular oxidative stress, they boost the cellular antioxidant defense systems through Nrf2, which regulates enzymes containing the antioxidant response element such as GPx or the gamma-glutamylcysteine synthetase involved in glutathione synthesis [70]. Therefore, it is possible that the increased GPx activity in the plasma of birds fed OE and SPICY might be a reflection of this hormetic effect. By contrast, the GPx activity was significantly lower in the plasma of birds fed the SPIOE compared to the PC.

Like vit E, the phytogenic additives had minor effects on the expression of target genes involved in the antioxidant response in the liver and jejunum. However, it is worth noting that the expressions of HSP70 and GSTA4 were significantly lower in the jejunum of chickens fed with OE compared to the NC. Heat shock proteins are expressed at low levels under normal physiological conditions, but their activity increases in response to a multitude of stressors, including oxidative stress [71]. The HSP70 plays a key role in oxidative stress signaling, and under this situation, its activation occurs to repair and adapt to redox changes. In this context, the use of antioxidants attenuates the activation of HSP70 by reducing the stress-causing agent [72]. Therefore, it is plausible that our result indicates a lower oxidative stress in the jejunum of birds fed the OE compared to those fed the NC. Polyphenols present in the OE have been reported to reduce HSP70 in heat-stressed Japanese quails [73]. As recently reviewed by Shehata et al. [74], decreased HSP70 in heat-stressed chicken after dietary consumption of different sources of plant extracts containing flavonoids, curcumin, or resveratrol is the consequence of their antioxidant capacity. Moreover, the olive triterpenes can up or down-regulate HSP70 expression. They can achieve that by directly binding to the gene nucleotide-binding domain of the HSP70 or indirectly through the inhibition of the transcription factor HSF1, which regulates its expression [75]. In line with this, the expression of GSTA4 was significantly lower in birds fed the OE compared to the NC. Glutathione S-transferases are a family of phase II detoxification enzymes that protect cells by conjugating glutathione with a broad range of electrophilic compounds of both endogenous and external origin [76]. The enzyme GSTA4 facilitates the removal of 4-hydroxynonenal (4-HNE), a potentially dangerous stable by-product of lipid peroxidation [76]. The lower expression of GSTA4 in this study conducted under normal oxidative stress conditions may indicate reduced lipid oxidation at the intestinal level in chickens fed with OE. Olive leaf extracts have been shown to decrease 4-HNE-induced phosphorylation of stress-activated proteins, including HSP27 [77]. Since there were no significant differences in Nrf2 expression, our results suggest that the OE extract has an antioxidant effect that is independent of Nrf2 and through the HSP70 signaling route. It has been reported that HSP70 modulates GPx activity [78], so the observed higher activity of this enzyme in the plasma of birds fed the OE compared to the NC might also be mediated through HSP70. Moreover, a high HSP70 expression has been shown to promote the production of pro-inflammatory cytokines [78]. Our previous studies with this OE showed an anti-inflammatory cytokine profile in the ileum of broiler chickens [23], suggesting that both the anti-inflammatory and antioxidant effects of the extract might be mediated through HSP70 signaling.

The finding of significant differences between the OE and SPICY diets in the expression of HSP70 and the tendency to decrease MDA concentrations later reinforces the OE antioxidant role and highlights the differences in the mode of action between the two extracts. Despite these differences between OE and Spicy, both extracts increased the plasma GPx activity compared to the NC. In the case of capsaicin, studies demonstrate that it has positive effects on glutathione, increasing the expression of GPx in ducks [79] and rats [68]. As previously indicated, one of the ways to explain the antioxidant effect of capsaicin is by producing a small prooxidant effect that results in indirect scavenging of ROS via Nrf2 [68]. This may explain the tendency toward a higher concentration of MDA and a greater activation of HSP70 compared to OE. However, we were unable to detect significant differences in Nrf2 expression among diets. Moreover, in our previous studies with the SPICY extract fed to broilers for 21 days of age, we observed reduced CAT activity in plasma and downregulation of CAT and Nrf2 genes in the liver compared to a control diet [60]. Both the age of the birds and the vit E concentration in the basal diet might be behind the discrepant results. Finally, the mix of both extracts maintained HSP70 expression to an intermediate value between OE and SPICY and significantly decreased the expression of GSTA4 compared to the NC. This might be indicative of the above-mentioned effects of OE through HSP70 signaling. However, the SPIOE decreased plasma GPx activity compared to the PC. All the changes found in antioxidant enzymes and genes may be indicative of the different mechanisms of action of the polyphenols, terpenoids, and alkaloids present in the two extracts and their mixtures, but their true significance will need to be addressed under stress conditions. Upcoming studies in which animals are subjected to common stressors in broiler production, such as heat stress, will provide new data on the mechanisms of action of these phytogenic additives.

## 5. Conclusions

The present study highlights the potential use of OE and SPICY extracts in diets with low vit E levels to maintain growth performance in the starter phase. Both phytogenic additives can sustain an antioxidant response mediated by GPx activity in comparison to a diet containing 100 ppm of vit E, even though this study’s environmental and nutritional conditions did not significantly increase oxidative stress and lipid peroxidation. The antioxidant effects of the OE seem to be mediated through the HSP70 signaling pathway, while those of the SPICY extract are not conclusive.

## Figures and Tables

**Table 1:** Ingredients, calculated and analyzed composition of the starter (0–21 d) and grower (21–35 d) basal diet.

Ingredient, %	Starter	Grower
Maize	58.7	64.0
Soybean meal (48%)	36.4	29.9
Soybean oil	1.20	3.05
Calcium carbonate	1.06	0.96
Monocalcium phosphate	1.02	0.50
Mineral/Vitamin premix ^1^	0.40	0.40
Sodium chloride	0.38	0.34
DL-Methionine	0.31	0.28
L-Lysine HCl	0.25	0.23
AxtraPhyt10000	0.10	0.10
L-Threonine	0.09	0.09
Monensin 20%	0.05	0.05
L-Valine	0.04	0.03
	Calculated composition	
AMEn ^2^, kcal/kg	2904	3087
Crude protein, %	21.64	19.08
Ash, %	5.65	4.70
Ether extract, %	3.82	5.72
Lysine, %	1.37	1.18
Methionine, %	0.63	0.57
Methionine + cystine, %	0.99	0.89
Threonine, %	0.91	0.81
Tryptophan, %	0.25	0.21
Calcium, %	0.87	0.72
Non phytic phosphorus, %	0.35	0.22
Sodium, %	0.16	0.16
	Analyzed composition	
Dry Matter, %	88.6	89.0
Gross energy, kcal/kg	3940	4052
Crude protein, %	22.8	19.7
Ash, %	6.03	4.88
Ether extract, %	4.02	5.42
Vit E, ppm ^3^	11.0	9.00

^1^ The premix provided per kg feed: vitamin A 10,000 UI; vitamin D3 4800 UI; vitamin B1 3 mg; vitamin B2 (riboflavin) 9 mg; vitamin B6 4.5 mg; vitamin B12 (cyanocobalamin) 40 μg; vitamin K3 3 mg; calcium pantothenate 16.5 mg; niacin 51 mg; folic acid 1.8 mg; biotin 0.15 mg; Fe (from FeSO_4_·H_2_O) 54 mg; I (from KI) 1.2 mg; Cu (from CuSO_4_·5H_2_O) 12 g; Mn (from MnSO_4_·H_2_O) 90 mg; Zn (from ZnO) 66 mg; Se (from Na_2_SeO_3_) 0.18 mg; BHT 25 mg; calcium carbonate as carrier up to 4 g. ^2^ AMEn, nitrogen-corrected apparent metabolizable energy. ^3^ The analyzed Vit E (alpha-tocopherol) concentrations for the positive control diet were 97 ppm for the starter diet and 104 ppm for the grower diet.

**Table 2:** Genes and primers for gene expression analysis by quantitative real-time PCR.

Gene *	5’-Primer Sequence Forward-3’	5’-Primer Sequence Reverse-3’	Reference
β-Actin	GTGATGGACTCTGGTGATGG	TGGTGAAGCTGTAGCCTCTC	[37]
UB	GGGATGCAGATCTTCGTGAAA	CTTGCCAGCAAAGATCAACCTT	[38]
CAT	GAGATGGTGAGGGCAGTTATT	GCCAATGTATGAGGAGGTTAGT	[39]
SOD1	TGGCTTCCATGTGCATGAAT	AGCACCTGCGCTGGTACAC	[40]
GPx1	CCACTTCGAGACCATCAAACT	GGTGCGGGCTTTCCTTTA	[39]
Nrf2	CAGAAGCTTTCCCGTTCATAGA	TGGGTGGCTGAGTTTGATTAG	[39]
NOX3	GAGTCCTGTGGTGCTGTATATC	GACTCCAGAGGAATGTGTTACC	[39]
HSP70	GGCTGGAGAGAAGAATGTGC	CAGCTGTGGACTTCACCTCA	[40]
GSTA4	TGCCACTGGTTGAGATCGACG	TCTCCTTTGCCTCAGGTGGA	[41]
GSTM2	GTGGACTTCCTGGCTTACGA	GCCGTGTACCAGAAAATGG	[42]
TTPA	TCCAGCAGTGGCCAAGAAAA	GCGAAGACTGGGTGGAAGAA	[43]

* β-Actin, beta actin; UB, ubiquitin; CAT, catalase; SOD1, superoxide dismutase; GPx1, Glutathione peroxidase 1; Nrf2, nuclear factor erythroid 2-related factor 2; NOX3, NADPH oxidase 3; HSP70, heat shock protein 70; GSTA4, glutathione S-Transferase Alpha 4; GSTM2, glutathione S-transferase Mu 2; TTPA, alpha tocopherol transfer protein.

**Table 3:** Effects of the experimental diets on the growth performance of the birds from 0 to 35 days of age ^1^.

		Treatments ^3,4^						Tukey, Adjusted *p*-Values		
Item ^2^		NC	PC	OE	SPICY	SPIOE	SEM (*n* = 8)	OE vs. SPIOE	SPICY vs. SPIOE	
**From 0 to 7 days**	BW0d, g	45.2	45.0	44.9	44.8	44.7	0.299	0.940	0.767	0.930
	BW7 d, g	194	195	191	196	194	2.84	0.191	0.554	0.744
	ADG, g/d	21.2	21.4	20.8	21.5	21.3	0.395	0.166	0.491	0.758
	ADFI, g/d	20.8	20.8	20.8	21.3	20.4	0.367	0.318	0.565	0.051
	FCR	0.984	0.967	0.999	0.987	0.963	0.018	0.782	0.121	0.361
**From 8 to 14 days**	BW14 d, g	505	522	506	517	517	7.82	0.345	0.311	0.997
	ADG, g/d	44.5	**46.7**	45.0	45.9	46.3	0.839	0.566	0.316	0.896
	ADFI, g/d	59.8	59.6	60.9	59.8	59.4	1.47	0.769	0.605	0.961
	FCR	1.35	1.28	1.35	1.30	1.28	0.038	0.465	0.213	0.861
**From 15 to 21 days**	BW21 d, g	1026	1063	1030	1051	1057	15.8	0.404	0.223	0.920
	ADG, g/d	74.3	77.4	74.9	76.3	77.1	1.64	0.685	0.391	0.876
	ADFI, g/d	98.3	98.3	101	98.9	99.1	3.07	0.751	0.783	0.998
	FCR	1.32	1.27	1.35	1.30	1.29	0.042	0.386	0.283	0.976
**Starter (from 0 to 21 days)**	ADG, g/d	46.7	48.5	46.9	47.9	48.2	0.753	0.400	0.217	0.917
	ADFI, g/d	59.1	60.1	60.9	60.0	60.0	1.49	0.803	0.676	0.970
	FCR	1.27	1.23	1.30	1.25	1.25	0.034	0.337	0.266	0.977
**From 22 to 28 days**	BW28 d, g	1803	1878	1803	1854	1816	34.6	0.313	0.925	0.518
	ADG, g/d	111	116	109	115	108	4.18	0.376	0.985	0.296
	ADFI, g/d	150	153	151	155	150	3.78	0.551	0.975	0.423
	FCR, g/d	1.35	1.32	1.41	1.35	1.39	0.052	0.560	0.970	0.706
**From 29 to 35 days**	BW35 d, g	2515	2644	2557	2611	2550	68.7	0.719	0.995	0.658
	ADG, g/d	102	109	108	108	105	7.66	0.999	0.928	0.911
	ADFI, g/d	173	175	174	175	172	5.19	0.988	0.934	0.870
	FCR, g/d	1.74	1.61	1.63	1.64	1.67	0.114	0.988	0.908	0.961
**Grower (from 22 to 35 days)**	ADG, g/d	106	113	108	111	107	4.59	0.793	0.923	0.561
	ADFI, g/d	161	164	162	165	161	4.06	0.829	0.943	0.636
	FCR	1.53	1.45	1.50	1.49	1.52	0.052	0.980	0.925	0.839
**Global (from 0 to 35 days)**	ADG, g/d	70.6	74.3	71.8	73.3	71.6	1.96	0.717	0.995	0.658
	ADFI, g/d	99.9	102	101	102	101	2.27	0.981	0.843	0.742
	FCR	1.40	1.20	1.41	1.39	1.42	0.113	0.981	0.996	0.994

^1^ Results are means ± standard error (n = 8 replicates, with 16 birds per replicate). ^2^ BW, body weight. ADG, average daily gain. ADFI, average daily feed intake. FCR, feed conversion ratio. ^3^ NC, negative control with no additives and no added vit E. PC, positive control with 100 ppm of supplemented vit E. OE, treatment with olive pomace extract. SPICY, treatment with capsicum, black pepper, and ginger extract. SPIOE, treatment with olive pomace extract and spicy extracts. ^4^ Results in bold are significantly different from NC, taken as reference, using a Dunnet adjusted test (*p* < 0.05).

**Table 4:** Effect of treatments on vitamin E concentrations and antioxidant enzyme activity in plasma ^1^.

	Treatments ^2,3,4^						Tukey, Adjusted *p*-Values		
Item	NC	PC	OE	SPICY	SPIOE	SEM (*n* = 8)	OE vs. SPICY	OE vs. SPIOE	SPICY vs. SPIOE
**α—tocopherol (ppm)**	*5.42*	30.6	*6.31*	*4.77*	*5.51*	1.91	0.698	0.906	0.920
**CAT ^5^ (U/mL)**	1.75	1.80	1.86	1.92	1.59	0.250	0.968	0.522	0.395
**SOD (U/mL)**	25.8	25.7	20.8	28.1	27.5	3.69	0.131	0.167	0.987
**GPx (mU/mL)**	506	**662**	537	564	*481*	61.2	0.893	0.633	0.367
**TBARs (μM MDA) ^6^**	0.296	0.325	0.243	0.418	0.294	0.074	0.051	0.789	0.225
**TAC (mM TE) ^7^**	2.66	2.46	2.51	2.19	2.92	0.503	0.792	0.702	0.335

^1^ Results are means ± standard error (*n* = 8 replicates). ^2^ NC, negative control with no additives and no added vit E. PC, positive control with 100 ppm of supplemented vit E. OE, treatment with olive pomace extract. SPICY, treatment with capsicum, black pepper, and ginger extract. SPIOE, treatment with olive pomace extract and spicy extracts. ^3^ Results in bold are significantly different from NC, taken as reference, using a Dunnet adjusted test (*p* < 0.05). ^4^ Results in cursive are significantly different from PC, taken as reference, using a Dunnet adjusted test (*p* < 0.05). ^5^ One unit of catalase is the amount of enzyme that will cause the formation of 1.0 nmol of formaldehyde per minute at 25 °C. One unit of superoxide dismutase is defined as the amount of enzyme needed to exhibit 50% dismutation of the superoxide radical measured in change in absorbance per minute at 25 °C and pH 8.0. One unit of glutathione peroxidase is defined as the amount of enzyme that causes the oxidation of 1.0 nmol of NADPH to NADP per minute at 25 °C. ^6^ TBARs = Thiobarbituric acid reactive substances, μM malondialdehyde. ^7^ TAC = total antioxidant activity, mM TROLOX equivalents.

**Table 5:** Effects of the experimental diets on the relative expression of selected genes in the jejunum mucosa ^1^.

	Treatments ^2,3^					Tukey, Adjusted *p*-Values		
Gene ^4^	NC	PC	OE	SPICY	SPIOE	OE vs. SPICY	OE vs. SPIOE	SPICY vs. SPIOE
**CAT**	1	0.854	0.775	1.03	1.02	0.267	0.300	0.997
	(1.20–0.835)	(1.02–0.713)	(0.928–0.647)	(1.23–0.860)	(1.22–0.849)			
**SOD**	1	1.29	1.15	1.08	1.14	0.884	0.999	0.898
	(1.14–0.879)	(1.47–1.14)	(1.31–1.01)	(1.23–0.947)	(1.30–1.01)			
**GPx1**	1	1.04	0.671	0.916	0.681	0.244	0.997	0.277
	(1.21–0.827)	(1.25–0.858)	(0.812–0.555)	(1.11–0.758)	(0.824–0.563)			
**Nrf2**	1	1.05	0.884	0.975	0.854	0.794	0.973	0.661
	(1.16–0.859)	(1.22–0.901)	(1.03–0.759)	(1.14–0.838)	(0.995–0.734)			
**HSP70**	1	0.727	**0.480**	0.920	0.599	0.0233	0.615	0.174
	(1.26–0.791)	(0.918–0.575)	(0.606–0.380)	(1.16–0.728)	(0.757–0.474)			
**GSTA4**	1	0.507	**0.188**	0.358	**0.295**	0.331	0.573	0.904
	(1.56–0.640)	(0.792–0.325)	(0.293–0.120)	(0.559–0.229)	(0.461–0.189)			
**GSTM2**	1	0.630	0.725	0.847	0.849	0.899	0.896	1.000
	(1.42–0.703)	(0.896–0.443)	(1.03–0.510)	(1.20–0.595)	(1.21–0.597)			

^1^ Relative gene expression values are fold change (2^−△△Ct^) of the experimental diets relative to the NC, which was set to be 1.0 (*n*  =  8 per treatment). Results are means, with values in brackets indicating the 95% confidence interval (Fold change up—Fold change low). ^2^ NC, negative control with no additives and no added vit E. PC, positive control with 100 ppm of supplemented vit E. OE, treatment with olive pomace extract. SPICY, treatment with capsicum, black pepper, and ginger extract. SPIOE, treatment with olive pomace extract and spicy extracts. ^3^ Results in bold are significantly different from NC, taken as reference, using a Dunnet adjusted test (*p* < 0.05). ^4^ CAT, catalase; SOD, superoxide dismutase; GPx1, glutathione peroxidase 1; Nrf2, nuclear factor erythroid 2-related factor 2; HSP70, heat shock protein 70; GSTA4, glutathione S-Transferase Alpha 4; GSTM2, glutathione S-transferase Mu 2.

**Table 6:** Effects of the experimental diets on the relative expression of selected genes in the liver ^1^.

	Treatments ^2^					Tukey, Adjusted *p*-Values		
Gene ^3^	NC	PC	OE	SPICY	SPIOE	OE vs. SPICY	OE vs. SPIOE	SPICY vs. SPIOE
**CAT**	1	0.995	0.890	0.798	0.985	0.767	0.799	0.386
	(1.17–0.855)	(1.17–0.851)	(1.04–0.761)	(0.934–0.682)	(1.15–0.841)			
**SOD**	1	1.09	1.22	1.12	1.09	0.815	0.711	0.983
	(1.15–0.868)	(1.25–0.946)	(1.41–1.06)	(1.28–0.972)	(1.26–0.948)			
**GPx1**	1	0.933	0.999	0.899	0.816	0.676	0.247	0.717
	(1.13–0.883)	(1.06–0.825)	(1.13–0.882)	(1.02–0.794)	(0.924–0.721)			
**Nrf2**	1	0.970	0.974	0.978	0.918	1.000	0.901	0.889
	(1.15–0.847)	(1.11–0.847)	(1.12–0.851)	(1.12–0.854)	(1.05–0.802)			
**NOX**	1	1.47	1.36	1.52	1.66	0.916	0.748	0.942
	(1.32–0.757)	(1.94–1.11)	(1.79–1.03)	(2.00–1.15)	(2.19–1.26)			
**HSP70**	1	0.748	0.617	0.746	0.790	0.627	0.456	0.957
	(1.23–0.815)	(0.918–0.609)	(0.757–0.503)	(0.916–0.608)	(0.970–0.644)			
**GSTA4**	1	0.938	0.977	0.914	0.810	0.942	0.631	0.825
	(1.23–0.816)	(1.15–0.766)	(1.20–0.797)	(1.12–0.746)	(0.993–0.661)			
**GSTM2**	1	0.936	0.933	0.953	0.981	0.99	0.942	0.98
	(1.16–0.860)	(1.09–0.805)	(1.09–0.802)	(1.11–0.819)	(1.14–0.843)			
**TTPA**	1	0.921	0.907	0.922	1.00	0.996	0.872	0.909
	(1.22–0.822)	(1.12–0.757)	(1.10–0.746)	(1.12–0.758)	(1.22–0.822)			

^1^ Relative gene expression values are fold change (2^−△△Ct^) of the experimental diets relative to the NC, which was set to be 1.0 (*n*  =  8 per treatment). Results are means, with values in brackets indicating the 95% confidence interval (Fold change up—Fold change low). ^2^ NC, negative control with no additives and no added vit E. PC, positive control with 100 ppm of supplemented vit E. OE, treatment with olive pomace extract. SPICY, treatment with capsicum, black pepper, and ginger extract. SPIOE, treatment with olive pomace extract and spicy extracts. ^3^ CAT, catalase; SOD, superoxide dismutase; GPx1, glutathione peroxidase 1; Nrf2, nuclear factor erythroid 2-related factor 2; NOX3, NADPH oxidase 3; HSP70, heat shock protein 70; GSTA4, glutathione S-Transferase Alpha 4; GSTM2, glutathione S-transferase Mu 2; TTPA, alpha tocopherol transfer protein.

## Data Availability

The data presented in this study are available upon request from the corresponding author.
